# Declining Economic Burden of Coronary Atherosclerosis Relative to the United States Economic Output and Healthcare Sector Growth

**DOI:** 10.7759/cureus.107924

**Published:** 2026-04-28

**Authors:** Aryan Gupta, Prabhat Gottipati, Priya Joshi, Alyster A Alcudia, Huaqing Zhao, Gregory Troutman, Yoshiya Toyoda, Suyog A Mokashi

**Affiliations:** 1 Division of Cardiovascular Surgery, Lewis Katz School of Medicine at Temple University, Philadelphia, USA; 2 Department of Biomedical Education and Data Science, Lewis Katz School of Medicine at Temple University, Philadelphia, USA

**Keywords:** atherosclerosis, coronary atherosclerosis, economic burden, healthcare spending, healthcare utilization, health economics, united states healthcare sector growth

## Abstract

Background

Coronary atherosclerosis remains a leading cause of ischemic heart disease and cardiovascular morbidity and mortality. While its clinical burden is well described, less is known about how its economic burden, defined using absolute expenditures and relative measures such as share of economic output, has changed over time.

Objective

To evaluate national trends in expenditures and utilization for coronary atherosclerosis and assess changes in its economic burden relative to the overall United States (US) economic output and healthcare sector growth.

Methods

National expenditure data from 2000 to 2021 were obtained from the National Income and Product Accounts (NIPA). Disease-specific spending was benchmarked against the total US gross output, used as a proxy for overall economic activity rather than direct gross domestic product (GDP) estimates, and the Healthcare and Social Assistance sector output. Outcomes included per capita spending, episode volume defined as aggregated counts of condition-specific healthcare encounters, total expenditures based on constant-dollar inflation-adjusted NIPA estimates, uncontrolled expenditures defined as non-population-adjusted totals, and cost per case. Temporal trends were evaluated using univariate linear regression.

Results

Between 2000 and 2021, per capita spending declined from $90.70 to $64.54 (−28.8%), with an average annual decrease of $1.86 (p<0.0001). Episode volume decreased from $13.34 million to $11.22 million (−15.9%), corresponding to an annual reduction of 136,874 episodes (p<0.0001). Total expenditures declined from $255.9 billion to $213.6 billion (−16.6%), with an annual decrease of $381.6 million (p=0.0003). Uncontrolled expenditures declined from $196.9 billion to $173.7 billion (−11.8%) (p=0.0004). Cost per case changed minimally, decreasing from $1,918 to $1,904 (less than 1% decline; p=0.026). Spending as a share of the total US economic output declined from 0.17% to 0.06% (p<0.001).

Conclusion

In this national, longitudinal analysis, the economic burden of coronary atherosclerosis declined across multiple absolute measures and relative benchmarks. These findings reflect reductions in spending and utilization over time; however, given the observational and aggregate nature of the data, underlying drivers cannot be determined. Macroeconomic benchmarking of disease-specific expenditures may provide a useful framework for evaluating long-term trends in healthcare spending.

## Introduction

Coronary atherosclerosis remains a leading cause of ischemic heart disease and cardiovascular mortality globally [1]. Despite sustained advances in medical therapy, interventional techniques, and public health initiatives, atherosclerotic cardiovascular disease (ASCVD) continues to drive substantial healthcare utilization, including hospitalization, procedural intervention, and long-term medical management, particularly in the context of an aging population [2].

While these challenges persist, the clinical landscape of ASCVD has evolved substantially over the past two decades. Population-level advances have included the widespread adoption of evidence-based lipid-lowering therapies, improved cardiometabolic risk management, anti-inflammatory strategies, and more refined risk-stratification approaches [3,4]. These developments have contributed to changes in patterns of care delivery, including earlier identification of high-risk patients and increased emphasis on outpatient management and prevention. In parallel, advances in cardiovascular imaging have enhanced the identification of high-risk plaque features and enabled monitoring of disease progression and therapeutic response [5]. Collectively, these innovations have been associated with substantial changes in ASCVD management and raise important questions regarding how these changes relate to healthcare utilization and economic burden. Accordingly, evaluating long-term trends in the economic burden of coronary atherosclerosis in relation to overall healthcare spending and economic growth may provide additional insight into these trends.

Prior studies have primarily examined trends in cardiovascular outcomes, utilization of emerging therapies, or payer-specific and cross-sectional costs associated with ASCVD [6,7]. However, the broader macroeconomic context in which these changes have occurred has not been well characterized, particularly with respect to benchmarking disease-specific expenditures against national economic indicators. Specifically, it remains unclear whether advances in ASCVD management are associated with a reduced economic burden when evaluated relative to overall economic growth and expansion of the healthcare sector. This distinction is important, as economic burden may be defined using absolute or relative measures, and changes in disease-related spending may reflect multiple factors, including population growth, inflation, and broader healthcare sector expansion. Assessing disease-specific expenditures in relation to national economic indicators may therefore provide a more informative framework for evaluating long-term healthcare system performance.

This analysis utilizes the National Income and Product Accounts (NIPA) database maintained by the Bureau of Economic Analysis (BEA) [8] to provide a data-driven, national-level framework for evaluating longitudinal healthcare expenditures within the United States (US) economy. Unlike claims-based datasets, which are limited to specific payers or provider networks, NIPA captures national spending across both public and private payers and enables direct comparison with gross output for all US industries, which serves as a proxy for overall economic activity rather than direct gross domestic product (GDP) estimates. Although NIPA provides a comprehensive, aggregate view of healthcare expenditures, disease-specific estimates are derived from categorized spending and may be subject to limitations in attribution granularity. By contextualizing disease-specific expenditures within broader economic and healthcare sector trends, NIPA data offer a useful framework for assessing whether the economic burden of coronary atherosclerosis has changed over time, both in absolute terms and relative to overall economic growth.

The primary objective of this study was to evaluate longitudinal trends in national expenditures and healthcare utilization associated with coronary atherosclerosis in the US from 2000 through 2021. Economic burden was defined using both absolute measures, including total and per capita expenditures, cost per case, and episode volume, and relative measures, including expenditures expressed as a proportion of overall economic output (gross output) and Healthcare & Social Assistance sector output. Secondary objectives included assessing changes in individual components of economic burden over time and benchmarking disease-specific expenditures against broader economic and healthcare sector growth. This analysis was designed to characterize temporal trends within a macroeconomic framework without inferring causality regarding underlying drivers.

This work was presented as a poster presentation at the 75th American College of Cardiology Annual Meeting in New Orleans, LA on March 28th, 2026.

## Materials and methods

This study employed a retrospective, longitudinal analysis of national healthcare expenditures from calendar years 2000 through 2021. Data were obtained from the NIPA maintained by the BEA [8]. NIPA captures aggregate national healthcare spending across public and private payers, enabling comparison of disease-specific expenditures with macroeconomic indicators, including overall economic output (gross output) and healthcare sector output. In contrast to claims-based databases, which are limited to specific insurers or provider networks, NIPA provides an economy-wide perspective suitable for aggregate-level economic benchmarking. This study used publicly available, de-identified, aggregate data and did not involve human subjects as defined by federal regulations. Institutional review board approval was not required.

Disease-specific spending was contextualized by benchmarking coronary atherosclerosis expenditures against total US economic output, measured using gross output, and against gross output for the Healthcare & Social Assistance sector, as defined by BEA industry classifications. Gross output was used as a proxy for overall economic activity to enable direct comparison with NIPA-derived expenditure estimates. These estimates represent aggregate national spending attributed to coronary atherosclerosis across the healthcare system. As NIPA is an aggregate expenditure dataset rather than a patient-level claims database, disease attribution is subject to limited clinical granularity and potential misclassification. Accordingly, expenditure estimates reflect condition-based spending at a population level and may not fully isolate coronary-specific pathology from related cardiovascular conditions. Coronary atherosclerosis expenditures were identified using the corresponding disease classification within the BEA Health Care Satellite Account (Agency for Healthcare Research and Quality/Clinical Classifications Software (AHRQ/CCS) category 101). Expenditure variables were reported in inflation-adjusted constant dollars using BEA chain-type price indices (base year as defined within the NIPA Health Care Satellite Account). In contrast, index-based measures (including per capita, medical care, and prevalence indices) were normalized to a base year of 2000 (index = 1.0) to facilitate relative comparisons over time. Within the NIPA framework, “episodes” represent aggregated counts of condition-specific healthcare encounters, and “uncontrolled expenditures” refer to total spending not adjusted for population size. 

Primary outcomes included per capita spending for coronary atherosclerosis, total NIPA expenditures, uncontrolled (non-population-adjusted) expenditures, and cost per case. Healthcare utilization was quantified using the annual number of coronary atherosclerosis-related episodes, as defined within the NIPA dataset and representing aggregated counts of condition-specific healthcare encounters. Cost per case was calculated as total disease-specific expenditures divided by the number of annual episodes. Uncontrolled expenditures refer to total aggregate spending without adjustment for population size, in contrast to per capita measures. Relative economic burden was assessed by expressing disease-specific expenditures as a proportion of the total US economic output (gross output) and total Healthcare & Social Assistance sector output. BEA datasets are subject to periodic revision, and the most recent available release at the time of analysis was used. No missing data were present for the variables included in this study.

Temporal trends for each outcome were evaluated using univariate linear regression, with calendar year specified as the independent variable. This approach was selected to characterize overall directional trends in spending and utilization over time in a consistent and interpretable manner. Regression slopes were used to estimate average annual changes in each outcome across the study period. Given the aggregate, longitudinal nature of the data, this analysis was intended to describe temporal associations rather than model causal relationships. While alternative modeling approaches, including non-linear or segmented regression, may capture potential inflection points or structural changes over time, the present analysis focused on linear trends for comparability across outcomes. Potential autocorrelation and structural changes over time were not formally modeled and represent a limitation of the analysis. Failure to account for autocorrelation may influence precision of regression estimates. Sensitivity analyses excluding 2020-2021 were not performed and represent an area for future study. Statistical significance was defined as a two-sided p value<0.05, and all analyses were performed using SAS 9.4 software (SAS Institute Inc., Cary, NC).

## Results

**Figure 1:**
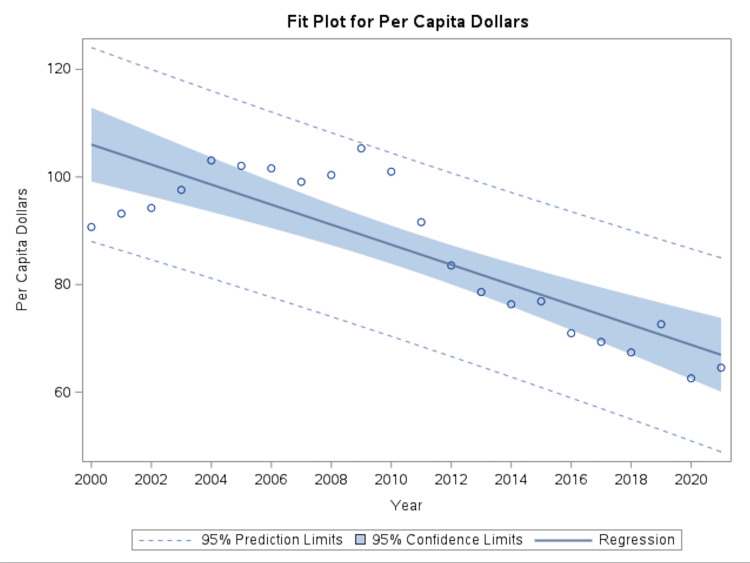
Temporal trends in per capita spending for coronary atherosclerosis Temporal trends in per capita spending for coronary atherosclerosis from 2000 to 2021 using the National Income and Product Accounts data. Per capita spending declined steadily over the study period. Solid line represents fitted univariate linear regression, with shaded areas denoting 95% CI.

Between 2000 and 2021, coronary atherosclerosis declined across major economic and utilization metrics. Per capita spending decreased from approximately $90.70 in 2000 to $64.54 in 2021, representing a 28.8% reduction based on observed values in 2000 and 2021. Linear regression analysis showed an average annual decrease of $1.86 per capita (p<0.0001; R² = 0.7063) (Figure 1).

**Figure 2:**
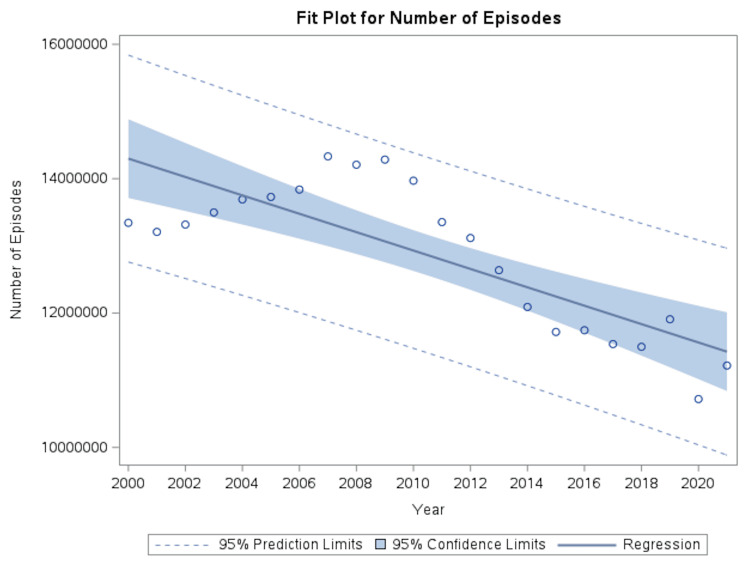
Temporal trends in annual coronary atherosclerosis episodes Annual number of coronary atherosclerosis-related episodes from 2000 to 2021 based on National Income and Product Accounts estimates. Episode volume demonstrated a consistent decline over time. Solid line represents fitted univariate linear regression with 95% CI.

Over the same period, healthcare utilization declined, with the annual number of coronary atherosclerosis-related episodes decreasing from approximately 13.34 million to 11.22 million, corresponding to a 15.9% reduction and an average annual decrease of 136,874 episodes (p<0.0001; R² = 0.6405) (Figure 2).

**Figure 3:**
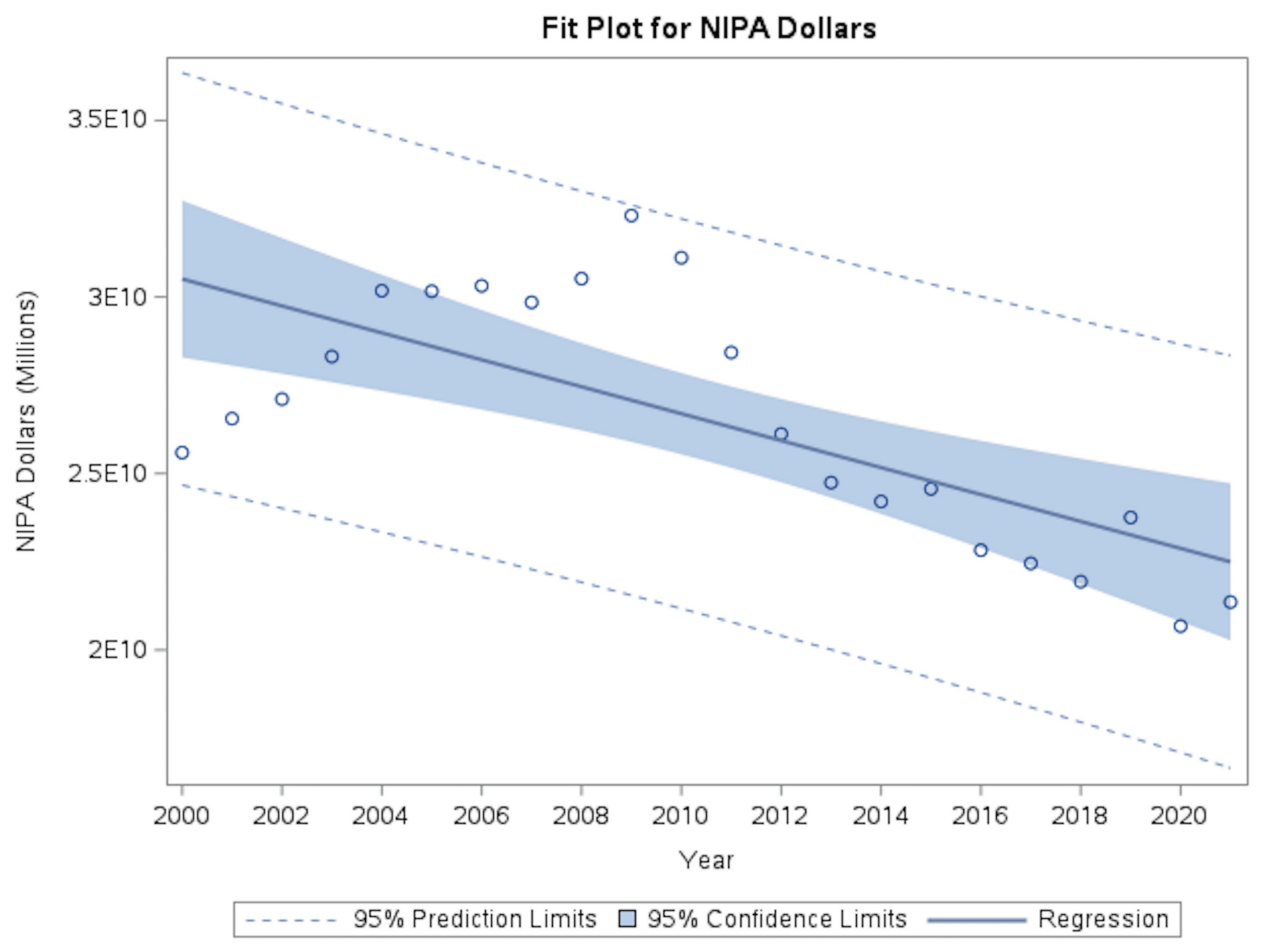
Temporal trends in national NIPA expenditures for coronary atherosclerosis Inflation-adjusted total national expenditures for coronary atherosclerosis from 2000 to 2021 using National Income and Product Accounts (NIPA) data. Expenditures are expressed in constant dollars and demonstrate a downward trend over time. Solid line represents fitted univariate linear regression with 95% CI.

Total national expenditures for coronary atherosclerosis decreased from approximately $255.9 billion in 2000 to $213.6 billion in 2021, representing a 16.6% reduction. Regression analysis demonstrated a mean annual decrease of $381.6 million (p=0.0003; R² = 0.4904) (Figure 3).

Uncontrolled expenditures similarly declined from approximately $196.9 billion to $173.7 billion, corresponding to an 11.8% reduction and an average annual decrease of $288.4 million (p=0.0004; R² = 0.4738). In contrast, cost per case changed minimally, decreasing from approximately $1,918 to $1,904 (<1% decline), with a small but statistically significant annual reduction of $8.36 per case (p=0.026; R² = 0.2233). Taken together, the larger decline in episode volume relative to cost per case suggests that reductions in total expenditures were primarily driven by decreased utilization rather than changes in cost intensity.

When evaluated relative to broader economic benchmarks, coronary atherosclerosis accounted for a smaller share of national and healthcare sector output. Disease-specific spending declined from approximately 0.17% of the total US economic output (gross output) in 2000 to 0.06% in 2021 (p<0.001). A similar pattern was observed when expenditures were benchmarked against total Healthcare & Social Assistance sector output, indicating sustained declines in relative economic burden over the study period.

## Discussion

In this national, longitudinal analysis, we found that the economic burden of coronary atherosclerosis declined in the US between 2000 and 2021. Reductions were observed across multiple dimensions, including per capita spending, episode volume, total national expenditures, and uncontrolled spending, indicating a consistent contraction in both individual-level and aggregate costs. These declines were also observed when benchmarked against overall economic growth and expansion of the healthcare sector, with coronary atherosclerosis accounting for a smaller share of both the US economic output and healthcare sector output over time. Together, these findings indicate that the relative economic footprint of coronary atherosclerosis has decreased over the past two decades, although the underlying drivers of these trends cannot be determined from this analysis.

Prior work has consistently demonstrated that cardiovascular disease represents a substantial component of the US healthcare spending, with ischemic heart disease alone accounting for tens of billions of dollars annually and total cardiovascular expenditures exceeding $250 billion in recent estimates [9]. While these estimates highlight the magnitude of cardiovascular spending in absolute terms, they do not account for concurrent growth in overall economic output or healthcare sector expansion, which may alter the relative economic burden over time. Given that cardiovascular disease continues to account for a substantial share of national healthcare spending, evaluating its trajectory relative to broader economic growth provides important context for assessing changes in healthcare utilization and resource allocation. In this context, the observed decline in relative economic burden in the present study suggests that growth in cardiovascular expenditures has not kept pace with broader economic and healthcare sector expansion.

The observed decline in the economic burden of coronary atherosclerosis may reflect several overlapping changes in cardiovascular care and healthcare delivery over the study period. Improvements in primary and secondary prevention, including broader use of lipid-lowering therapies and more effective management of cardiometabolic risk factors, are potential contributors and are consistent with prior trends in cardiovascular care [3]. In parallel, advances in guideline-directed medical therapy and risk-based stratification may have influenced patterns of care delivery and resource utilization [4]. However, given the aggregate and ecologic nature of the data, alternative explanations must also be considered. Changes in coding practices, shifts in site of care from inpatient to outpatient settings, and evolving patterns of pharmaceutical management may influence measured expenditures without necessarily reflecting true reductions in disease incidence or severity. In addition, broader healthcare system factors, including reimbursement changes and care delivery reforms, may contribute to observed spending patterns. As such, the mechanisms underlying the observed decline in economic burden cannot be directly determined from this analysis and should be interpreted as hypothesis-generating rather than causal.

Utilization of coronary atherosclerosis-related care declined in parallel with overall spending over the study period. In this analysis, reductions in total expenditures were primarily driven by decreases in episode volume, while cost per case remained relatively stable. This pattern suggests that changes in utilization, rather than reductions in per-episode costs, contributed most to the observed decline in spending. Although shifts in care delivery, including evolving site-of-care patterns and more selective use of interventions, may influence resource utilization, these factors cannot be directly assessed within the present dataset [6]. Similarly, advances in risk stratification and guideline-directed therapy may contribute to changes in utilization patterns, but their impact cannot be quantified in this analysis [4]. Accordingly, the observed trends are best interpreted as reflecting changes in aggregate utilization rather than direct evidence of improved efficiency or reduced care intensity.

Evaluating disease-specific expenditures relative to broader economic and healthcare sector growth provides important context that absolute spending measures alone cannot capture. Absolute trends may obscure meaningful system-level changes by failing to account for inflation, population growth, and the expansion of the healthcare sector over time. By benchmarking coronary atherosclerosis spending against overall economic output (gross output) and Healthcare & Social Assistance sector output, this analysis provides a normalized assessment of how the economic footprint of this condition has evolved within the US economy [10]. The observed contraction in relative burden indicates that spending on coronary atherosclerosis has grown more slowly than both the overall economy and the healthcare sector. However, as gross output was used as a proxy for economic activity rather than direct measures of GDP, these comparisons should be interpreted with caution. Such macroeconomic benchmarking may offer a complementary perspective for evaluating long-term trends in disease-specific spending across the healthcare system [11]. Gross output reflects total economic activity rather than value-added GDP. Therefore, these comparisons should be interpreted as approximations of relative economic burden rather than direct GDP-equivalent estimates.

These findings have important implications for value-based cardiovascular care and health system planning. The declining relative economic burden of coronary atherosclerosis suggests that changes in prevention, early intervention, and care delivery may be associated with observed changes in spending patterns. As healthcare systems increasingly emphasize cost containment and value, understanding trends in disease-specific spending can help inform resource allocation and long-term planning [12]. Moreover, continued monitoring of expenditures in relation to broader economic growth may aid policymakers and healthcare leaders in identifying areas where targeted investment may influence future healthcare utilization and spending patterns [13].

This study should be interpreted in the context of several limitations. First, the analysis is ecologic in nature and relies on aggregate, national-level data, precluding patient-level inference or causal conclusions regarding drivers of observed trends. Second, expenditure estimates derived from the NIPA data reflect broad spending categories and may not capture granular variation in care delivery, payer mix, or regional practice patterns, and are subject to potential misclassification in disease attribution. Third, although trends in spending and utilization were evaluated over time, this analysis did not assess clinical outcomes or disparities, which may evolve differently across populations. Fourth, the use of gross output as a proxy for overall economic activity, rather than direct measures of GDP, may influence interpretation of relative economic burden. Fifth, this analysis focuses on coronary atherosclerosis and does not capture non-coronary manifestations of ASCVD, which may result in underestimation of the broader economic burden of ASCVD. Finally, inclusion of the COVID-19 period (2020-2021) may introduce variability related to temporary disruptions in healthcare utilization and delivery that cannot be fully disentangled within this framework.

## Conclusions

In this national, longitudinal analysis, the economic burden of coronary atherosclerosis declined across multiple absolute and relative measures between 2000 and 2021. Reductions in per capita spending, total expenditures, and healthcare utilization were accompanied by a marked contraction in disease-specific spending relative to both overall US economic output and healthcare sector growth, indicating a sustained decrease in the relative economic footprint of coronary atherosclerosis within the broader economy. The fact that cost per case remained largely stable while overall expenditures declined further indicates that reductions in utilization, rather than changes in care intensity, were the primary driver of these trends.

Evaluating disease-specific spending within a macroeconomic framework offers a useful perspective on healthcare system performance and may help inform future research and resource allocation strategies in cardiovascular care. Of course, the observed trends may reflect a combination of true reductions in disease burden, changes in coding and care delivery, and broader systemic factors such as reimbursement structures and healthcare policy shifts. Overall, this study underscores the importance of evaluating disease-specific expenditures within a macroeconomic framework. Future work integrating patient-level outcomes, payer-specific data, and regional variation will be essential to better define the drivers of these trends and to inform resource allocation.
